# Analysis of cerebral infarction caused by dysplasminogenemia in three pedigrees

**DOI:** 10.3389/fgene.2023.1132654

**Published:** 2023-03-30

**Authors:** Xuanyu Chen, Ming Zou, Chunxing Lu, Ruyi Zhou, Shuyue Lou, Yujia Wang, Hongxiang Ding, Zhao Han, Beilei Hu

**Affiliations:** ^1^ Department of Neurology, Second Affiliated Hospital and Yuying Children’s Hospital of Wenzhou Medical University, Wenzhou, China; ^2^ Clinical Laboratory, Second Affiliated Hospital and Yuying Children’s Hospital of Wenzhou Medical University, Wenzhou, China

**Keywords:** dysplasminogenemia, juvenile stroke, plasminogen, genetic mutations, heredity

## Abstract

**Background and aims:** Dysplasminogenemia is a rare heritable disease caused by plasminogen (PLG) gene defects resulting in hypercoagulability. In this report we describe three notable cases of cerebral infarction (CI) complicated with dysplasminogenemia in young patients.

**Methods:** Coagulation indices were examined on STAGO STA-R-MAX analyzer. PLG: A was analyzed using a chromogenic substrate-based approach using a chromogenic substrate method. All nineteen exons of PLG gene and their 5′and 3′flanking regions were amplified by Polymerase chain reaction (PCR). Suspected mutation was confirmed by reverse sequencing.

**Results:** PLG activity (PLG:A) in proband 1 and 3 of his tested family members, proband 2 and 2 of his tested family members, and proband 3 and her father were all reduced to roughly 50% of normal levels. Sequencing led to the identification of a heterozygous c.1858G>A missense mutation in exon 15 of the PLG gene in these three patients and affected family members.

**Conclusion:** We conclude that the observed reduction in PLG:A was the result of this *p*.Ala620Thr missense mutation in the PLG gene. The CI incidence in these probands may be attributable to the inhibition of normal fibrinolytic activity as a consequence of this heterozygous mutation.

## 1 Introduction

Plasminogen (PLG) is a single-stranded glycoprotein that is primarily produced in the liver prior to secretion into systemic circulation in an inactive zymogen form (Pons et al., 2016). PLG is encoded by 52.5 kb gene on chromosome 6q26-q27 comprised of 19 exons and 18 introns. The 2.7 kb PLG coding sequence code for a 791 amino acid protein (Xia et al., 2019). Functionally, PLG is a key component of the plasma fibrinolytic system that can be converted into plasmin by specific enzymatic activators, thereby driving fibrin (Fb) degradation to facilitate the dissolution of intravascular thrombi (Castellino and Ploplis, 2005). There are two primary subtypes of PLG deficiency. In type I PLG deficiency, also known as hypoplasminogenemia, PLG activity (PLG:A) and PLG antigen (PLG:Ag) levels are decreased, whereas in type II PLG deficiency, also known as dysplasminogenemia, PLG:A levels are decreased despite normal PLG:Ag levels (Cheng et al., 2016). Of the two, dysplasminogenemia is more frequently associated with thrombotic events. Cerebral infarction (CI) is the cause of 60%–80% of stroke incidence, occurring due to the localized ischemic necrosis of brain tissue as a result of an insufficient blood supply, ultimately causing neurological defects. Affected patients suffer from high disability and mortality rates (Liu and Liu, 2020). Further advances in molecular biology techniques have advanced our understanding of the association between serological characteristics and CI. In this report, we describe the phenotypic and genetic characteristics of three young CI patients and family members affected by dysplasminogenemia, in addition to exploring the factors linking dysplasminogenemia and CI.

## 2 Participants and methods

### 2.1 Patients

#### 2.1.1 Family A

The proband in Family A was a 16-year-old Chinese male that was admitted to The Second Affiliated Hospital and Yuying Children’s Hospital of Wenzhou Medical University due to a 1-day history of sudden onset right limb weakness. Neurological examination revealed that the right limb exhibited normal muscle tone and reflexes, with a strength scale score of 4/5. Right-sided ataxia was less coordinated than that on the left, and the patient exhibited right-sided Babinski sign positivity. No sensory disturbances were detected. Cerebral magnetic resonance imaging (MRI) revealed evidence of an acute infarction with left lateral ventricle involvement. Cardiac examination and ultrasonographic inspection of the cervical and lower extremity vasculature did not reveal any obvious abnormalities. Laboratory test results were normal, including blood counts, renal and hepatic function results, blood glucose levels, lipid levels, and thyroid function. Other test results included D-dimer (D-D) 0.62 mg/L, PLG:A 65%, F VIII:C 210%, vWF:Ag 136.0%. No obvious abnormalities were apparent among other measured indices, and further details are provided in Table 1.

**TABLE 1:** Phenotype and genotype test results of three pedigrees with inherited dysplasminogenemia.

Family members	Ages (years)	Ala620Thr c.1858G>A	Plasma concentration							
			PC:A (%)	PS:A (%)	AT:A (%)	FIB (mg/L)	D-D (mg/L)	FDP (mg/L)	PLG:A (%)	PLG:Ag (%)
Pedigree 1										
**Proband (Ⅲ**_**1**_ **)**	**16**	**Heterozygote**	**78**	**79**	**97**	**2.57**	**0.62**	**3.7**	**65**	**84**
Grandpa (Ⅰ_1_)	77	Heterozygote	86	75	96	3.85	0.47	2.3	54	77
Grandma (Ⅰ_2_)	76	Normal	98	82	99	2.36	0.34	2.8	97	112
Father (Ⅱ_1_)	54	Heterozygote	87	86	101	3.12	0.21	1.9	53	89
Mother (Ⅱ_2_)	50	Normal	104	93	112	2.74	0.19	2.1	89	103
Sister (Ⅲ_2_)	28	Heterozygote	96	88	98	3.13	0.32	2.6	51	88
Pedigree 2										
**Proband(Ⅲ**_**1**_ **)**	**14**	**Heterozygote**	**95**	**67**	**99**	**3.99**	**0.71**	**2.7**	**56**	**78**
Grandpa (Ⅰ_1_)	70	Heterozygote	120	77	100	2.11	0.78	3.2	43	82
Grandma (Ⅰ_2_)	66	Normal	123	90	106	3.01	0.42	3.3	99	76
Uncle (Ⅱ_1_)	40	Normal	101	84	103	3.14	0.33	1.4	115	94
Aunt (Ⅱ_2_)	37	Normal	96	102	114	2.63	0.25	2.3	98	103
Mother (Ⅱ_3_)	34	Heterozygote	114	96	107	3.01	0.26	2.1	49	114
Father (Ⅱ_4_)	36	Normal	87	83	116	2.13	0.24	1.9	112	78
Pedigree 3										
**Proband(Ⅱ**_**1**_ **)**	**19**	**Heterozygote**	**96**	**89**	**107**	**2.36**	**1.71**	**3.4**	**58**	**86**
Father (Ⅰ_1_)	46	Heterozygote	107	96	98	3.01	0.48	2.1	52	92
Mother (Ⅰ_2_)	45	Normal	102	91	104	2.98	0.32	1.9	98	85
Reference range			70–130	65–135	96–118	2.0–4.0	0–0.5	0–5.0	80–120	70–140

PC:A: protein C activity; PS:A: protein S activity; AT:A: antithrombin activity; FIB: fibrinogen; FDP: fibrinogen degradation product; PLG:A: plasminogen activity; PLG:Ag: plasminogen antigen. The bold values indicate the phenotypeand genotype test results of probands.

#### 2.1.2 Family B

The proband in Family B was a 14-year-old Chinese male admitted to The Second Affiliated Hospital and Yuying Children’s Hospital of Wenzhou Medical University for a 2-day history of left-sided limb weakness. Physical examination results were normal, and MRI scans revealed an acute infarction involving the right pontine infarct. As with proband A, cardiac examination and ultrasonographic inspection of the cervical and lower extremity vessels did not reveal any abnormal findings. Laboratory test results were normal, including blood counts, renal and hepatic function results, blood glucose levels, lipid levels, and thyroid function. Other test results included D-D 0.71 mg/L, PLG:A 56%, F VIII:C 142%, vWF:Ag 116.0%.

#### 2.1.3 Family C

The proband in family C was a 19-year-old Chinese female admitted to The Second Affiliated Hospital and Yuying Children’s Hospital of Wenzhou Medical University for an over 10-day history of a headache when lying flat and an intracranial infarction detected 5 days previously. The results of neurological examinations were normal, while MRI scans revealed cerebral venous sinus thrombosis and acute cerebral infarction. Cardiac examination and ultrasonographic inspection of the cervical and lower extremity vessels did not reveal any abnormal findings. Laboratory test results were normal, including blood counts, renal and hepatic function results, blood glucose levels, and lipid levels. Other test results included D-D 1.71 mg/L, PLG:A 58%, FⅧ:C 209%, vWF: Ag 170.0%. No obvious abnormalities were observed for other indices.

### 2.2 Blood clotting analyses

In total, 100 healthy individuals from 23 to 47 years of age (42 females, 58 males) were selected to establish a normal reference for coagulation phenotype indices. These healthy controls were free of any apparent liver or kidney dysfunction, thrombophilia, or spontaneous bleeding tendency. The Ethics Committee of The Second Affiliated Hospital and Yuying Children’s Hospital of Wenzhou Medical University of China approved this study, and all participants and appropriate family members provided written informed consent.

### 2.3 Material and methods

1. Data collection: Clinical data collection and pedigree analyses were performed for three CI patients.
2. Sample collection and processing: After having obtained informed consent from patients and their family members, peripheral blood samples were collected in vacuum tubes (containing 1:9 0.109 M trisodium citrate). Samples were centrifuged for 10 min at 2,100 xg, and the upper platelet-poor plasma fraction was collected for measurements of coagulation indices while the pelleted blood cells were used to isolate genomic DNA for sequencing.
3. Plasma coagulation index analyses: Fibrinogen (FIB), F VII:C, and protein S activity (PS:A) were analyzed *via* a one-stage clotting approach. D-D, vWF:Ag, and fibrinogen degradation products (FDP) were measured *via* immunoturbidimetry. PLG:A, antithrombin activity (AT:A), and protein C activity (PC:A) were analyzed using a chromogenic substrate-based approach using a chromogenic substrate method. PLG:Ag levels were measured *via* enzyme-linked immunosorbent assay (ELISA).
4. Genetic testing: Genomic DNA from enrolled patients and family members was extracted from peripheral blood leukocytes based on DNA Extraction Kit (Tiangen, Beijing, China), after which DNA concentration and purity were measured with a DU800 protein nucleic acid spectrophotometer (Beckman Corporation, United States). PCR amplification of target sequences was performed *via* PCR using primers and thermocycler settings published previously (Cheng et al., 2016). Primers were synthesized by Sunsoon BIO-Technology Corporation, Shanghai, China.

After PCR products had been purified, they were sequenced by Sunsoon BIO-Technology Corporation (Shanghai, China). Sequencing results generated using the Chromas software were compared to National Center for Biotechnology Information (NCBI) reference PLG gene sequence (Genbank AY192161.1). Reverse sequencing was used to confirm identified variant sites in probands, and family members then underwent sequencing to determine whether they were carriers for these same variants.

## 3 Results

### 3.1 Clinical features

**FIGURE 1:**
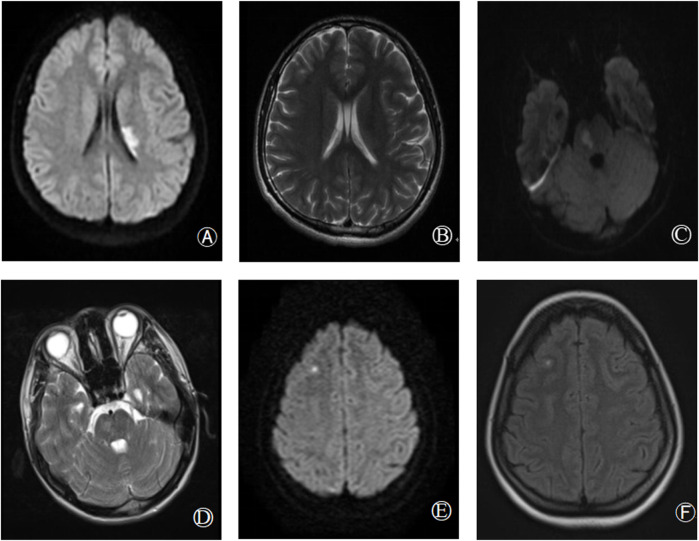
Brain imaging findings of hereditary abnormal plasminogen patients [proband 1: **(A,B)**; proband 2: **(C,D)**; proband 3: **(E,F)]**. **(A,B)**: Diffusion weighted imaging (DWI) and T2 sequence respectively showed acute infarction in left paraventricular; **(C,D)**: DWI and T2 respectively showed acute infarction in right pons; **(E,F)**: DWI, T2 sequence, respectively showed acute infarction in the right frontal lobe.

Genotypic and phenotypic analysis results for all study participants are detailed in Table 1, with probands marked in bold. The PLG:A levels of probands 1, 2, and 3 were respectively reduced to 65%, 56%, and 58% of normal levels (reference range: 80%–120%), whereas their PLG:Ag levels were within normal ranges. The PLG: A levels of the grandfather, father, and sister of proband 1, the grandfather and mother of proband 2, and the father of proband 3 were all reduced to ∼50% of normal levels. Sequencing results revealed that all of these individuals carried the identified mutation, whereas other family members were not carriers for this mutation and exhibited normal PLG:A and PLG: Ag levels. All family members exhibited normal PC, PS, and antithrombin activity. MRI scans for probands 1, 2, and 3 revealed instances of acute infarction in the left paraventricular, right pons, and right frontal cortex regions, respectively (Figure 1).

### 3.2 DNA sequence analysis

**FIGURE 2:**
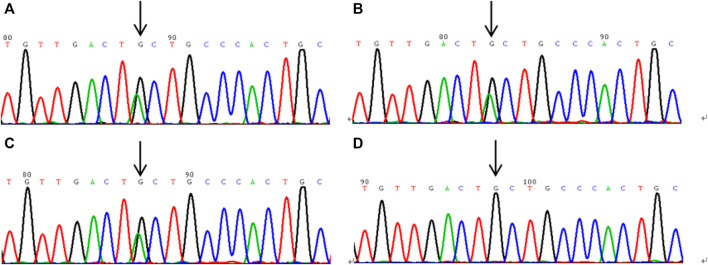
The sequencing results of plasminogen gene in exon 15 of hereditary plasminogen deficiency patients. **(A–C)**: the gene sequencing results of probands 1, 2 and 3, respectively, and the heterozygous mutation site c.1858G > **(A)** is shown by the arrow. **(D)** is the gene sequencing result of the wild type.

The sequencing results are shown in Figure 2. Gene analysis showed the proband 1 and his 3 family members, proband 2 and his 2 family members, proband 3 and her father had the heterozygous mutation of c.1858G>A in exon 15, which resulted in a mutation of alanine at position 620 in PLG to threonine (*p*.Ala620Thr).

## 4 Discussion

CI is a common clinical disease that is associated with high morbidity, mortality, and disability rates. While CI is most common in adults over 50 years of age, juvenile stroke rates are also rising, with an estimated incidence of 8.7–11.3 per 100,000 (Gong et al., 2020). Unlike in older patients, juvenile stroke cases are often the result of a wider range of relatively rare risk factors such as impaired coagulation, patent foramen ovale (PFO), or non-atherosclerotic vascular disease, with the actual cause being undetermined in many cases. Abnormal coagulation is thought to contribute to ∼4% of all juvenile stroke cases (Majid et al., 2020). All three patients discussed in this report were young. After having excluded common CI-related risk factors, these patients were assessed for coagulation indices including AT:A, PC:A, PS:A, PLG:A, and PLG:Ag levels, of which only PLG:A was significantly reduced. Accordingly, these three patients were tentatively diagnosed with dysplasminogenemia, and subsequent PLG gene sequencing led to the identification of the heterozygous Ala620Thr mutation in all three patients.

Dysplasminogenemia is a rare autosomal disease that exhibits incomplete dominance, resulting in non-specific clinical presentations that can range from asymptomatic to thrombotic disease (Cheng et al., 2016). The question as to whether PLG deficiency is a relevant thrombotic risk factor remains open. In 1978, Aoki et al. (1978) published the first case report of dysplasminogenemia-related thrombosis, with the affected patient exhibiting a PLG:A level of just 40%. However, Okamoto et al. (2003) found through human studies that cases of both heterozygous and homozygous dysplasminogenemia due mainly to PLG-Ala620Thr are not related to deep vein thrombosis. Through animal studies, Tashima et al. (2017) found that the Ala620Thr mutation, does not increase the risk of thrombotic diseases in mice. Other reports have conversely identified arterial thrombosis as a main risk facing patients with dysplasminogenemia. Further studies of 77 CI patients led Masao et al. (Nagayama et al., 1996) to determine that PS deficiencies and PLG abnormalities were common among young CI patients, potentially contributing to the incidence of this condition. In another 1993 analysis of 3 young CI patients, Nagayama et al. (1993) determined that dysplasminogenemia is a major risk factor related to the risk of ischemic cerebrovascular disease among younger individuals, in line with the findings published by Kato et al. (1999) based on the evaluation of a 10-year-old CI patient. These findings highlight dysplasinemia as a major cause of CI. In line with these reports, the present analysis of three families identified the presence of the heterozygous p.Ala620thr mutation, consistent with dysplasminogenemia, in all three probands. These probands were also free of other known CI-related risk factors, suggesting a link between CI incidence and dysplasminogenemia.

Plasminogen is the primary component of the fibrinolytic system, helping to limit inappropriate fibrin deposition. Reductions in PLG:A activity contribute to the inhibition of fibrinolytic function, increasing the risk of thrombosis (Iwaki et al., 2010). Singh et al. (2016) determined that elevated PLG levels were sufficient to protect against ischemic brain injury, with these protective benefits largely being mediated through the reduction of microvascular thrombosis. The Ala620Thr mutations may not only cause the haploinsufficiency of the plasminogen protein, but may also alter its activity by functioning as an inhibitor of some plasminogen function. The p.Ala620Thr mutation is common in Asian populations. A multiple sequence alignment comparison revealed p.Ala620 to be highly conserved across 11 homologous versions of this protein, suggesting it is a major functional site in the PLG protein necessary for its normal activity (Cheng et al., 2016; Xia et al., 2019). This mutation is likely to be deleterious, altering protein function as confirmed through four predictive bioinformatics tools (Mutation Taster, PolyPhen-2, PROVEAN, and SIFT). Mutant protein model analyses revealed that this p.Al620Thr mutation resulted in a change in the internal protein structure such that it was abnormally formed with decreased catalytic activity. The American College of Medical Genetics and Genomics also indicated that the p.Ala620Thr missense mutation should be classified as pathogenic (Richards et al., 2015).

While associations between plasminogen deficiency and CI incidence remain uncertain, these results indicate that patients affected by dysplasminogenemia may face a higher risk of CI as compared to healthy controls. As such, routine analyses of PLG:A should be performed in juvenile and young adult CI patients, particularly when these patients are free of relevant risk factors. Establishing the etiological basis for CI is central to defining an appropriate treatment plan and preventing recurrent disease. Anticoagulant therapy is generally the most common treatment in patients exhibiting thrombophilia with dysplasminogenemia (Nagayama et al., 1993). However, whether this treatment strategy is optimal remains uncertain, and further case reports will be required to reliably summarize disease-related prognostic outcomes.

In conclusion, we herein determined that the observed reductions in PLG:A in probands and their relatives were likely the result of the p.Ala620Thr missense mutation in the PLG gene. Decreased PLG:A contributes to the inhibition of fibrinolytic function, thereby increasing the risk of thrombosis. The CI incidence in these probands may be attributable to impaired fibrinolytic activity due to the identified p.Ala620Thr missense mutation. However, the study sample size was limited, and further large-scale research exploring the link between hereditary dysplasminogenemia and CI will be necessary to firmly establish this relationship.

## Data Availability

The original contributions presented in the study are included in the article/supplementary materials, further inquiries can be directed to the corresponding author.
